# The Sacred Mountain of Varallo in Italy: Seismic Risk Assessment by Acoustic Emission and Structural Numerical Models

**DOI:** 10.1155/2013/170291

**Published:** 2013-12-05

**Authors:** Alberto Carpinteri, Giuseppe Lacidogna, Stefano Invernizzi, Federico Accornero

**Affiliations:** Department of Structural, Geotechnical and Building Engineering, Politecnico di Torino, 10129 Torino, Italy

## Abstract

We examine an application of Acoustic Emission (AE) technique for a probabilistic analysis in time and space of earthquakes, in order to preserve the valuable Italian Renaissance Architectural Complex named “The Sacred Mountain of Varallo.” Among the forty-five chapels of the Renaissance Complex, the structure of the Chapel XVII is of particular concern due to its uncertain structural condition and due to the level of stress caused by the regional seismicity. Therefore, lifetime assessment, taking into account the evolution of damage phenomena, is necessary to preserve the reliability and safety of this masterpiece of cultural heritage. A continuous AE monitoring was performed to assess the structural behavior of the Chapel. During the monitoring period, a correlation between peaks of AE activity in the masonry of the “Sacred Mountain of Varallo” and regional seismicity was found. Although the two phenomena take place on very different scales, the AE in materials and the earthquakes in Earth's crust, belong to the same class of invariance. In addition, an accurate finite element model, performed with DIANA finite element code, is presented to describe the dynamic behavior of Chapel XVII structure, confirming visual and instrumental inspections of regional seismic effects.

## 1. Introduction: The Historic Site of the Sacred Mountain of Varallo

**Figure 1:**
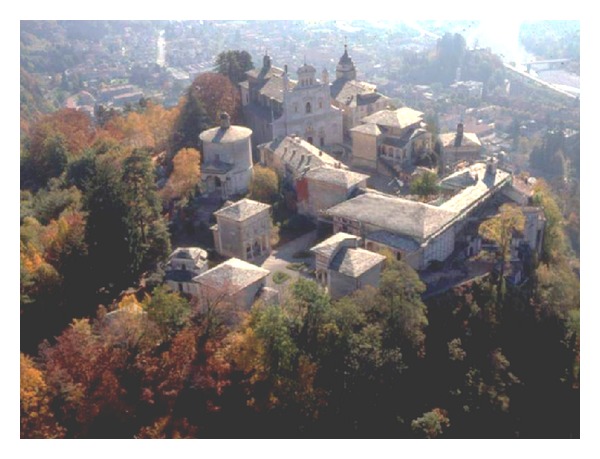
The Sacred Mountain of Varallo, Italy. Overview.

The Sacred Mountain of Varallo is located in the Italian province of Vercelli. Built on a cliff above the town of Varallo, it is the oldest and most important Sacred Mountain of the Alps (Figure 1). This historical site is composed of a Basilica and 45 Chapels, some of which are isolated, while others are part of monumental groups. The Chapels contain over 800 life-size wooden and multicoloured terracotta statues, which represent the Life, Passion, and Death of Christ. The site is considered the most notable example in the group of Sacred Mountains of Piedmont, a complex that has been included in the UNESCO World Heritage List since 2003.

Its story began in the late fifteenth century when the Franciscan friar Bernardino Caimi of Milan, returning from the Holy Land where he was guardian of the Holy Sepulchre, decided to reproduce in Varallo the holy places of Palestine [1].

**Figure 2:**
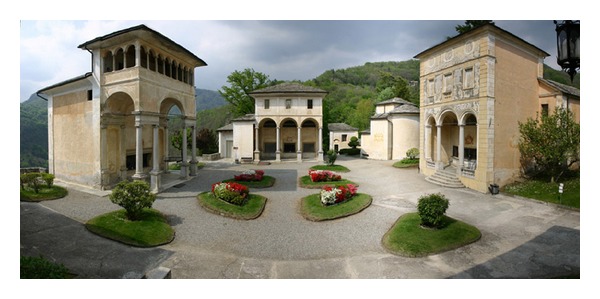
The Sacred Mountain of Varallo. The Square of Tribunals.

**Figure 3:**
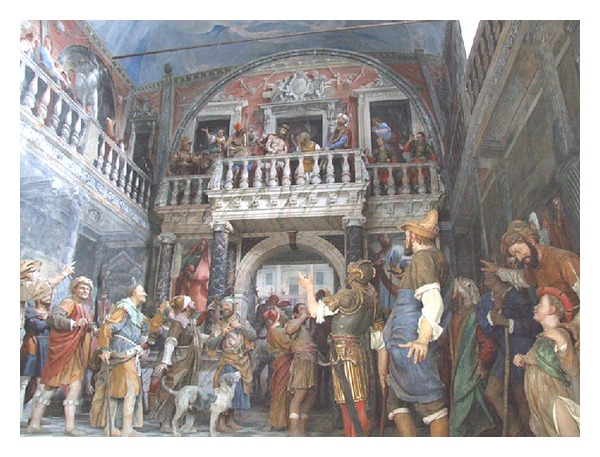
Chapel XXXIII. *Ecce Homo*.

The “New Jerusalem,” as it was called the Sacred Mountain, initially intended to represent the distant sites of the Christian tradition for all those people who could never go there (Figure 2). Inside these places are, instead of pictures, paintings and sculptures to evoke the corresponding event in the history of the life of Christ. Already in the early sixteenth century, thanks to the work of the painter sculptor and architect Gaudenzio Ferrari, the scenes inside the chapels are represented in an ingenious and innovative merging of painting and sculpture, with a strong sense of reality, so that the devotee could feel himself deeply involved by the depicted scene of the Christ's Life and almost part of it (Figure 3). The work of Gaudenzio Ferrari will be taken as a model in the construction of many other Sacred Mountains. In the era of the Counter-reformation, the Sacred Mountain of Varallo assumed the appearance of a path, real but at the same time mystical, that the pilgrim completes following the telling of the story of Christ's Life [1].

## 2. Chapel XVII Monitoring Set-Up

**Figure 4:**
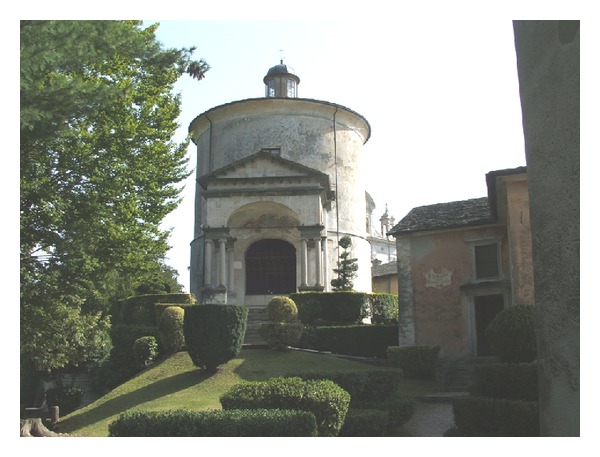
Chapel XVII. *The Transfiguration of Christ on Mount Tabor*.

The Acoustic Emission monitoring was conducted on the frescoed masonry walls of the Chapel XVII of the Sacred Mountain of Varallo: the Chapel of the Transfiguration of Christ on Mount Tabor (Figure 4). The construction of the Chapel XVII began in 1572, but the structure was completed only in 1647. In 1664 the lantern top was built as crowning.

One of the purposes of monitoring by means of AE sensors applied to the frescoed wall was to detect the AE signals from a region of the wall in which the frescos show a plaster detachment. Moreover, we used the collected data coming from the “in situ” monitoring in order to assess the seismic risk of artworks and possible collapses due to earthquake actions [2, 3].

**Figure 5:**
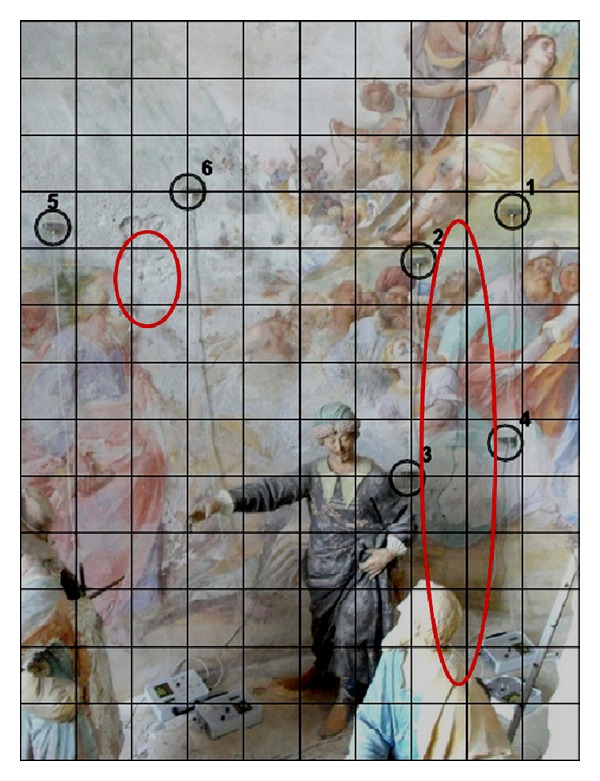
Chapel XVII. View of the monitored areas. Left side: sensors 5, 6, and the frescos detachment. Right side: sensors 1–4 and the vertical crack.

As regards the structural integrity, the North wall of the Chapel XVII shows a vertical crack of about 3.00 m in length and a detachment of frescos. These phenomena were one of the objective of the monitoring campaign by means of AE technique. Six AE sensors were employed to monitor the damage evolution of the structural support of the decorated surfaces: four were placed around the vertical crack while two were positioned near the frescos detachment (Figure 5). In addition, the South wall of the Chapel XVII also shows a vertical crack, symmetric to the previous one with respect to the pronaos of the building.

For the sensor pasting on decorated surfaces, a suitable methodology was applied. The necessary operations for bonding the AE sensors to the wall were carried out by a group of restorers, which have prepared a film of Japanese paper, on the surface of which is coated a thin layer of “Paraloid.”

The “Paraloid” is an acrylic resin (methyl acrylate soluble in ketones, esters, hydrocarbons, and chlorinated hydrocarbons) and is used in the field of restoration as a consolidant at low concentrations (2,4%) or as an adhesive at higher concentrations. It allows an excellent waterproof performance and has the advantage of being reversible and long-term stable. The layer of “Paraloid” forms a good protective base for the AE sensors bonding with silicone glue. The sensors were applied to monitor both the vertical crack and the detachment of the plaster (Figure 5) [2, 3].

**Figure 6:**
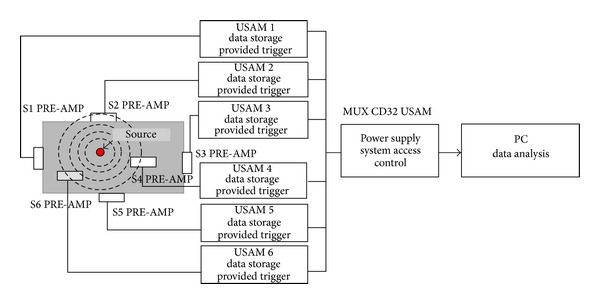
AE acquisition system.

The Acoustic Emission acquisition system is shown schematically in Figure 6. The piezoelectric transducers (PZT) are calibrated over a range of frequency between 50 kHz and 800 kHz. The USAM acquisition system consists of 6 preamplified sensors, 6 units of data storage provided of triggers, a central unit for the synchronization operations, an internal clock, and a trigger threshold. The obtained data are the cumulative counting of each mechanical wave considered as acoustic emission events, the acquisition time, the measured amplitude in Volt, the duration, and the number of oscillations over the threshold value for each wave [4, 5].

The monitoring time started from May 9, 2011, and finished on September 5, 2011. Regarding the monitoring results on the chapel structural integrity, they are reported in [2]. We interpreted the AE data by means of statistical analysis, considering the amplitude and time distribution of AE signals during the cracking phenomena. From this analysis, we found that the vertical crack monitored on the North wall of the chapel is in a stable condition during the acquisition period, while the process of detachment of the frescos is evolving cyclically. It appears that the frescos degradation could be mainly related to the diffusion of moisture in the mortar substrate [2].

## 3. AE and Seismic Events

Nondestructive testing methods are currently used to evaluate structural damage phenomena and to predict their development over time. It is worth noting that the evaluation of damage in historic buildings is often a complex task. It is essential to distinguish between stable damage patterns and damage in evolution towards a catastrophic collapse. Some structural damage can be triggered by events such as earthquakes. Furthermore, the limited ductility of the masonry, combined with the large size of this type of construction, provides a rather fragile structural behavior. Fortunately, the damage evolution in time can be effectively evaluated by means of the AE technique [4–10].

Moreover, the statistical distribution of earthquakes shows a complex nonlinear space-time behavior, that reflects the heterogeneities of the Earth's crust. Despite this complexity, a scaling law is universally valid: the earthquakes frequency-magnitude statistical distribution provided by the Gutenberg-Richter (GR) law [11].

On the other hand, AE in materials and earthquakes in the crust are very similar from many aspects and correlated in time, even though they occur at very different scales [12]. In both cases, there is a release of elastic energy from a source located in the medium, respectively, the tip of opening microcracks and the seismic hypocenter [13]. This similarity suggests that the seismic events and the AE events can be related in space and time. Therefore, it is possible to search for a correlation between the AE parameters related and the regional seismicity. In our opinion, this approach can be used to identify some warning signals that anticipate a catastrophic collapse of a structure. In fact, in many cases, the warning signals can be detected well in advance with respect to the time at which the unrecoverable damage event will occur [14, 15].

Most earthquakes have precursors, that are phenomena that in the short or long term change their activity before the earthquake. In the literature, many precursors have been proposed, but there is still no clear evidence about their reliability. In addition, any operative warning procedure must be based on the acquisition of a combination of several precursor clues. Recently, major efforts in the field of earthquake prediction have focused on the fluctuations of the physical parameters of the crustal rocks of the seismically active continental areas, and on regular intervals in the space-time distribution of earthquakes [16]. The variation in the rate of the regional seismicity is considered as a precursor in the long term. A region which had a small earthquake activity for a remarkable number of years is called “seismic gap.” The “seismic gaps” are considered as potential sites for major earthquakes. On the other hand, the increasing pressure in the rock surface in the region of the epicenter produces numerous cracks before the final collapse and, as a result, it causes changes in the properties of rocks. Therefore, the drop in speed of seismic waves caused by the expansion of the rock becomes a significant precursor. Other precursors linked to the expansion of the rocks and the opening of cracks are the crustal tilt and elevation changes, the decrease of the electrical resistivity of the rocks, and the release of radon gas in the atmosphere, which requires small pores to propagate. As the process of damage develops, the water diffuses from the surrounding rocks in pores and microcracks of increasing size, which in the meantime are forming. The moment the water fills the cracks, the speed of seismic waves grows, the soil lifting stops, the emission of radon from the new cracks is relieved, and the electrical resistivity decreases. The next stage is the beginning of the earthquake, which is immediately followed by several aftershocks in the surrounding area [7, 16].

When a crack in the Earth's crust increases (i.e., a fault propagate), the corresponding AE show, progressively lower frequencies, eventually decreasing from the ultrasound field down to the sonic range: as it occurs during the well-known phenomenon of seismic roar. Thus, AE techniques can be effectively put into relation with the spread of tensions through the Earth's crust. Some Italian researchers collected continuously, for many years, the AE signals from below the Gran Sasso massif [14, 15]. The progressive decrease of detected AE frequencies reveals that the damage localization is taking place, while AE high-frequency can be associated with the increase of small lesions more uniformly distributed in the crust. Therefore, the potential of earthquake prediction related to the AE monitoring appears promising [14].

### 3.1. Correlation Algorithms between AE and Seismic Events

Among the various studies on the earthquakes space-time correlation, there is a statistical method that allows to calculate the degree of correlation both in space and time between a series of AE and the local seismic recordings, collected in the same period. This analysis is based on the generalization of the space-time correlation known as the integral of Grassberger-Procaccia [17], defined as follows:

(1) $$\begin{array}{c} C\left(r,\tau \right)\equiv \frac{1}{N_{\text{EQ}}N_{\text{AE}}}\sum_{k=1}^{N_{\text{EQ}}}\;\sum_{j=1}^{N_{\text{AE}}}\Theta \left(r-\left|x_{k}-x_{j}\right|\right)\Theta \left(\tau -\left|t_{k}-t_{j}\right|\right), \end{array}$$

where *N*_AE_ is the number of peaks of AE activity registered in site, in this case Chapel XVII, and in a defined time window, *N*_EQ_ is the number of earthquakes recorded in the surrounding area during the same time window and Θ is the step function of Heaviside (Θ(*x*) = 0 if *x* ≤ 0, Θ(*x*) = 1 if *x* > 0). The index *k* refers to the recorded seismic events {*x*_*k*_, *t*_*k*_}, while the index *j* refers to the recorded AE events {*x*_*j*_, *t*_*j*_}.

Therefore, between all possible pairs of recorded AE and seismic events, the sum expressed by the integral of Grassberger-Procaccia can be calculated for those having the epicentral distance |*x*_*k*_ − *x*_*j*_| ≤ *r* and the temporal distance |*t*_*k*_ − *t*_*j*_| ≤ *τ*. Hence, *C*(*r*, *τ*) is the probability of occurrence of two events, an earthquake and an AE event, whose mutual spatial distances are smaller than *r* and mutual temporal distances are smaller than *τ*.

Note that, in order to evaluate (1), the numbers of *N*_AE_ and *N*_EQ_ are not required to assume the same value, and that *x*_*j*_ correspond to the geographic position of the Chapel.

Anyway, this approach does not consider the chronological order of the two types of events. Since the AE time series and the earthquake sequences are closely intertwined in the time domain, the problem of the predictive ability of the AE peaks is still open. The records of AE could be both the consequences of the progressive development of microdamage or the effect of widespread microseismicity. Therefore, a probabilistic analysis can be carried out discriminating between the AE events prior to the earthquake, which are precursors, and the AE following the earthquake, which are aftershocks. This analysis can be performed adopting a modified correlation integral [7] as follows:

(2) C±(r,τ)≡1NEQNAE∑k=1NEQ∑j=1NAEΘ(r−|xk−xj|)Θ  ×(τ−|tk−tj|)Θ(±(tk−tj)),

where “+” and “−” in the Heaviside function are used to take into account that the AE events could be, respectively, seismic precursors and aftershocks.

**Figure 7:**
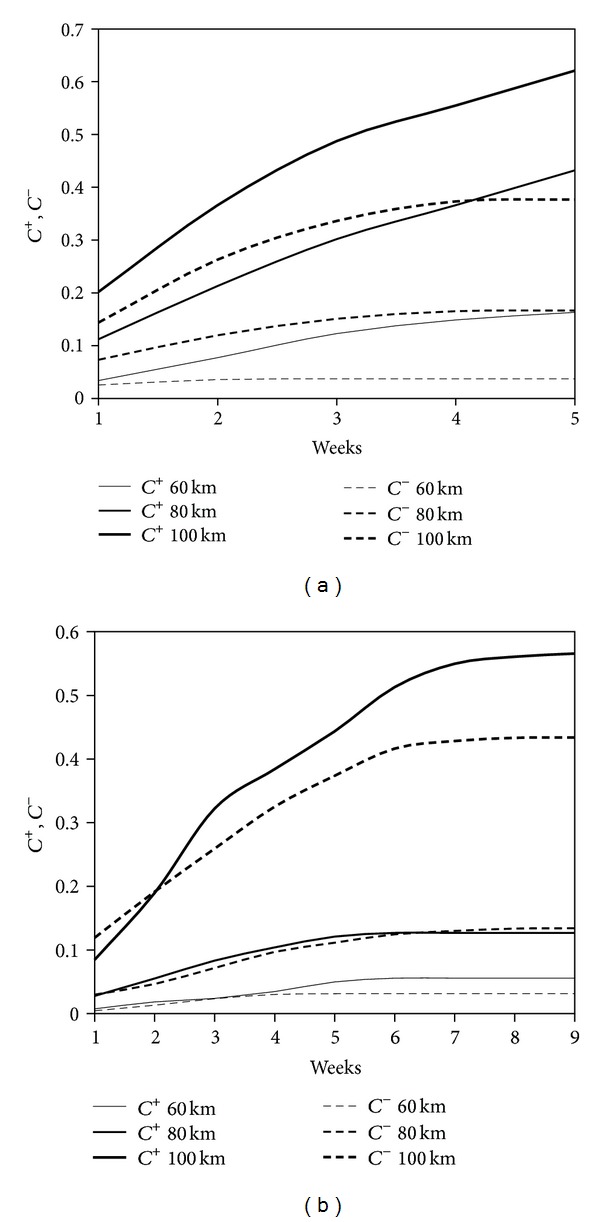
Evolution of the modified correlation integral for different time windows, during the two monitoring period. (a) Monitoring time from May 9, 2011 to June 16, 2011, see Tables 1 and 2. (b) Monitoring time from July 5, 2011 to September 5, 2011, see Tables 5 and 6.

In this way, the function *C* ^+^(*r*, *τ*) gives the probability that a peak of AE, detected at a certain time, will be followed by an earthquake in the subsequent days within a radius of *r* kilometers from the AE monitoring site. Varying the thresholds *r* and *τ* in (2), two cumulative probability distributions can be constructed, one for each condition (sign “+” or “−”) and then the corresponding probability density functions can be derived and represented (see Figure 7 and Tables 1–6, in which the radius *r* ranges between 60 and 100 kilometers, and the time interval *τ* varies from 1 week up to a maximum of 9 weeks).

## 4. AE as Seismic Precursors in the Sacred Mountain of Varallo

### 4.1. AE Monitoring Periods

For this analysis, the AE collected data are grouped into two different time windows. The first time window started May 9, 2011, and finished June 16, 2011. The second time window started July 5, 2011, and finished September 5, 2011. Both time windows involved the monitoring of the vertical crack and of the frescos detachment [2].

### 4.2. Recognizing Impending Earthquakes by means of AE

In this section, we obtain a correlation between seismic and acoustic events through the application of the modified integral of Grassberger-Procaccia.

**Figure 8:**
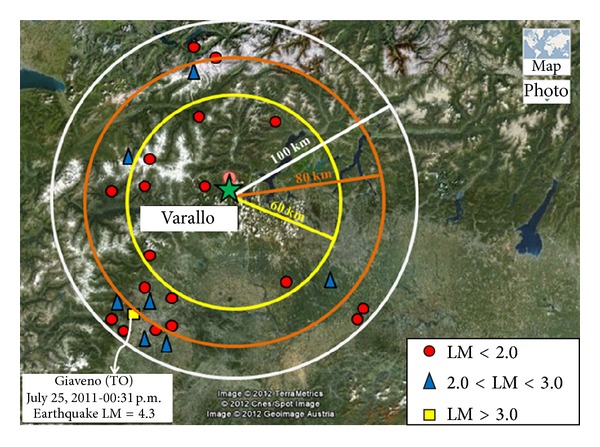
Seismic events around Varallo (Italy) from May, 2011 to September, 2011 (LM: local magnitude).

**Figure 9:**
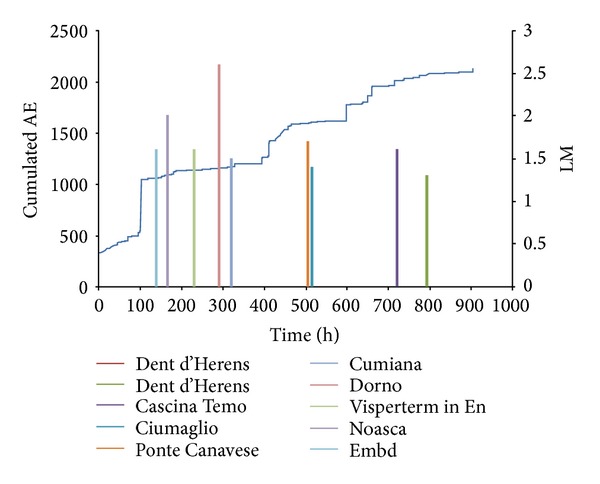
Sacred Mountain of Varallo: cumulated AE and seismic events from May 9, 2011 to June 16, 2011 (LM: local magnitude).

The data series of analyzed AE are shown in Figures 8 and 9, and are related to the abovementioned time intervals. The seismic events are taken from the website http://iside.rm.ingv.it/iside/standard/result.jsp?rst=1&page=EVENTS#result (seismic catalog of INGV, National Institute of Geophysics and Volcanology), selecting the events comprised in a circle of 100 km radius around the site of the Sacred Mountain of Varallo, during the defined AE monitoring periods (Figure 8).

**Figure 10:**
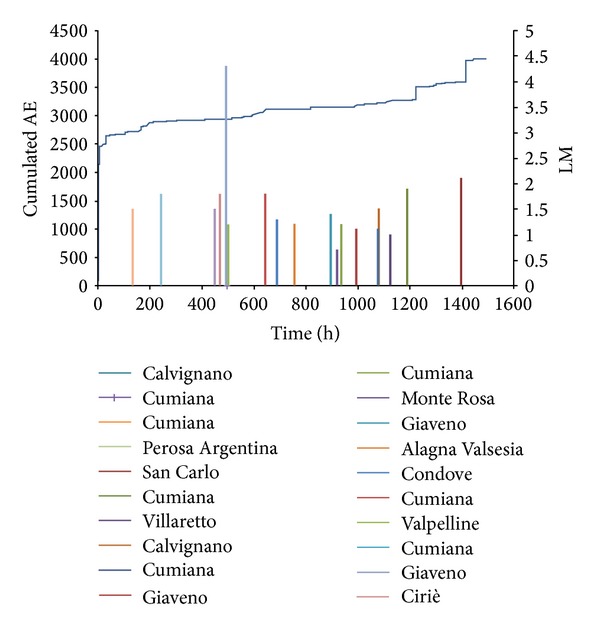
Sacred Mountain of Varallo: cumulated AE and seismic events from July 5, 2011 to September 5, 2011 (LM: local magnitude).

Looking at the temporal distribution of earthquakes in relation to the cumulative AE trend, a quite good correspondence between AE peaks and earthquake events can be observed (Figures 9 and 10). By applying the modified correlation integral of Grassberger-Procaccia to the data series, we obtain the cumulative probabilities, as a function of the radius of interest *r* and of the interval of occurrence *τ*, both considering the peak of Acoustic Emission as earthquake precursor or as aftershock (Tables 1–4).

The probability values obtained for the period May-June show that, regardless of the distance and of the correlation time, the probability of a seismic event following a peak of Acoustic Emission (AE *Precursor*) is always greater than the probability of the same AE peak being an effect of the damage caused by the earthquake (AE *Aftershock*) (Tables 1 and 2). In practice, we see that the monitored structure behaves as a sensitive seismic receptor.

It is interesting to note that, for both monitoring periods, within a radius of 60 km from the monitored site, the AE signals still play their role as seismic precursors, as it can be assessed observing the values of the cumulative probability *C* ^+^. On the contrary, at distances of 80 km and 100 km, the character of AE occurrence is different (see Tables 3 and 4). In particular, we observe a clear reversal of the AE signal character from precursor to aftershock for the second monitoring period (July–September).

More in detail, within a radius of 60 km, there is a clear tendency of AE signals to anticipate earthquakes, and behave as precursors. At a distance of 80 km, AE are precursor signals only in the time window comprised between 2 and 6 weeks, were *C* ^+^ > *C* ^−^. At a distance of 100 km, all the AE signals behave as aftershocks, being always *C* ^+^ < *C* ^−^.

In any case, it is worth distinguishing between the environmental contributions due to crustal trembling (external source), and the structural damage contributions (inner source) to AE activity on the Chapel XVII.

To better analyze the results from the second monitoring period (Tables 3 and 4), it is useful to discriminate the recorded signals assigning thresholds both in frequency and amplitude, which are consistent with the physical nature of Acoustic Emissions detected by the sensors. Looking at the USAM data stored, a good choice to discriminate the recorded signals is setting a frequency threshold equal to 30 kHz, which divides the field VLF (Very Low Frequency) from the field LF (Low Frequency), and a signal amplitude threshold equal to 1 mV. The choice of the thresholds (amplitude and frequency) was based upon empirical considerations in order to emphasize the signals coming farther from the structure, which were characterized by lower frequencies and amplitudes.

The results are shown in Tables 5 and 6, where it can be easily recognized that filtered AE signals actually behave like seismic precursor. From a theoretical point of view, lower frequencies allow for the diffusion of the elastic waves in the masonry bulk, either intact or damaged, while higher frequency waves can propagate only through small heterogeneities [18–20]. Moreover, if constant velocity is assumed, the Lamb ratio [21] implies that the AE wavelength has to be larger than the size of the maximum inhomogeneity in order to travel through the ground up to the structure without significant modifications in its waveform [22]. On the other side, low amplitudes are reasonably related to the fact that an event captured by AE sensors on the monitored structure may have originated from a source that is physically distant from the monitored site (surrounding microseismicity that shakes the whole structure) and therefore is subject to the laws of amplitude damping [20].

## 5. Finite Element Modeling: Spectral Dynamic Analysis of the Chapel XVII

**Figure 11:**
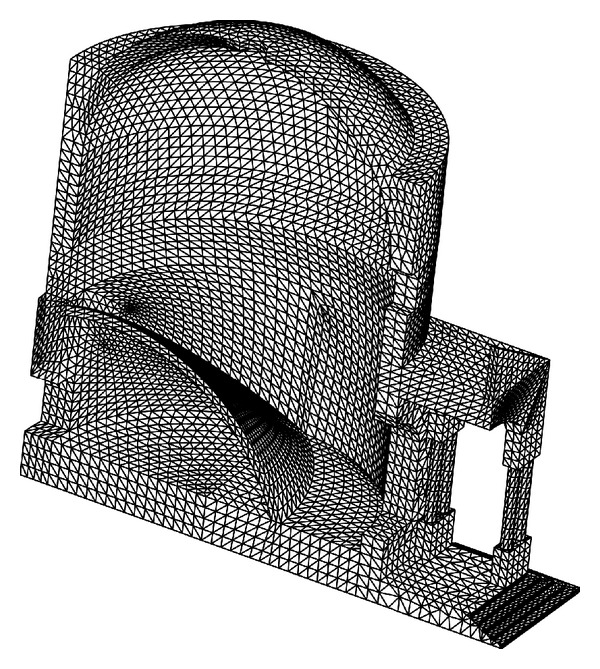
Finite element mesh of Chapel XVII: half of the model.

**Figure 12:**
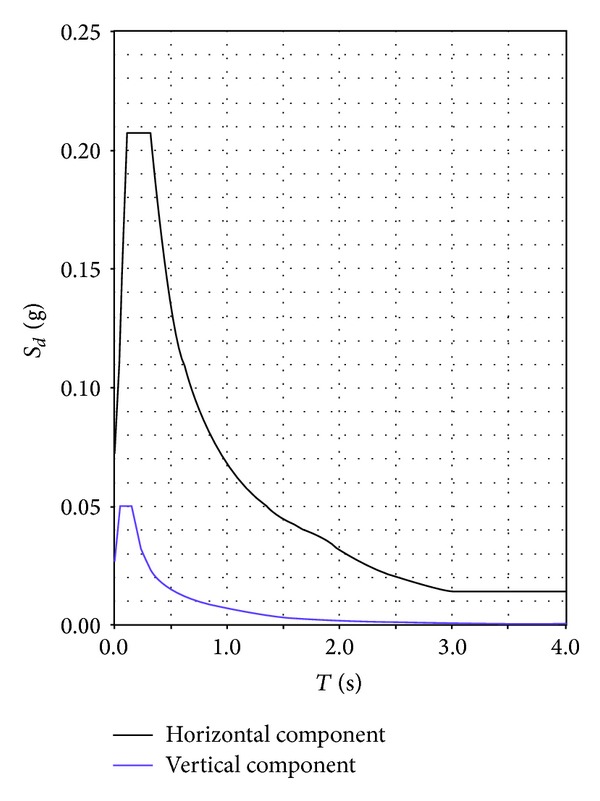
Elastic response spectrum of acceleration *S*_*d*_ (g).

The Chapel XVII was discretized with three-dimensional linear pyramid elements, accounting for the accurate geometry of the stone masonry structure. The shapes of the cylindrical chapel and of the above spherical dome are precisely discretized, taking into account the various apertures, the inside internal vault supporting the Mount Tabor installation, and the outside pronao with columns. On the contrary, the wooden roof structure was considered only as an external load. The mesh of the structure is shown in Figure 11. The finite element model is discretized using 30271 nodes, connected by 129689 elements, and is characterized by 86076 degrees of freedom. The elastic properties assumed for the masonry and the density, where, respectively, equal to *E* = 2 × 10^9^ Pa; *ν* = 0.3; *γ* = 20 kN/m^3^. For the dynamic analysis, the Elastic Response Spectrum of Acceleration *S*_*d*_ (g) is shown in Figure 12. It is obtained considering the geographic coordinates of the site of investigation, the soil characteristics, a nominal life of the structure equal to 400 years, and a probability to exceed the acceleration spectrum equal to 5% in 400 years. The structural damping of masonry is considered to be equal to 5%. The vertical component of the Spectrum is neglected [23].

**Figure 13:**
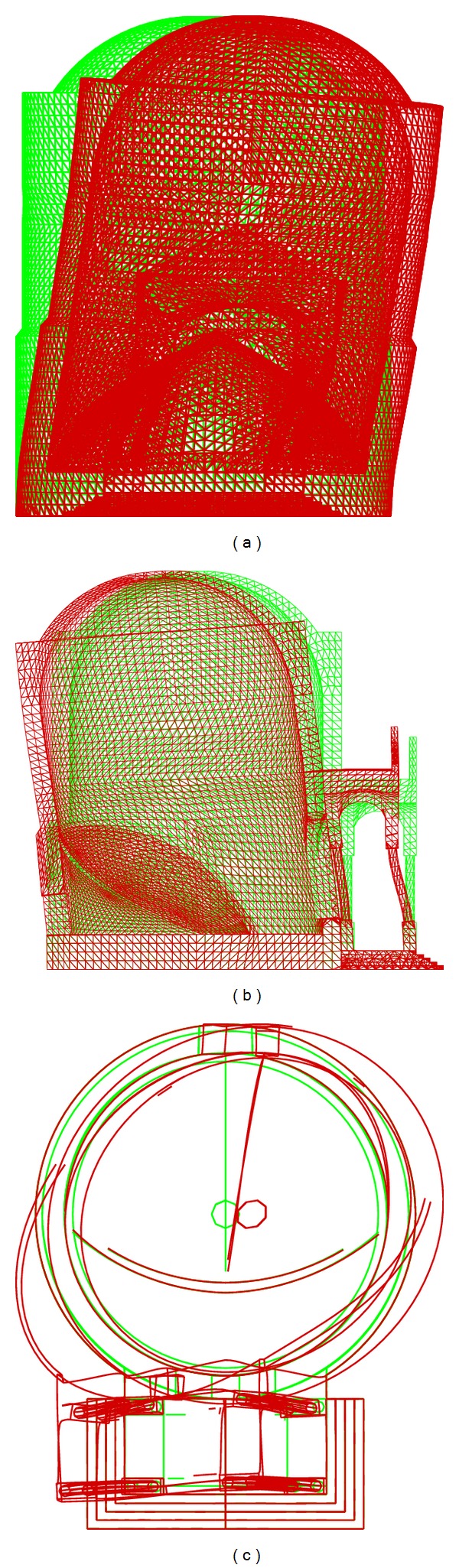
Chapel XVII mode 1 of vibration (a); Chapel XVII mode 2 of vibration (b); Chapel XVII mode 3 of vibration (c).

The dynamic analysis, performed with the commercial finite element code DIANA [24] allows for a preliminary assessment of the modal behavior of the structure.  Table 7 shows natural frequencies and periods of the first 20 calculated modes of vibration of Chapel XVII, and percentages of mass involved in each mode of vibration for directions *X* and *Y* in the horizontal plane. The first 3 modes of vibration of the structure are shown in Figure 13.

**Figure 14:**
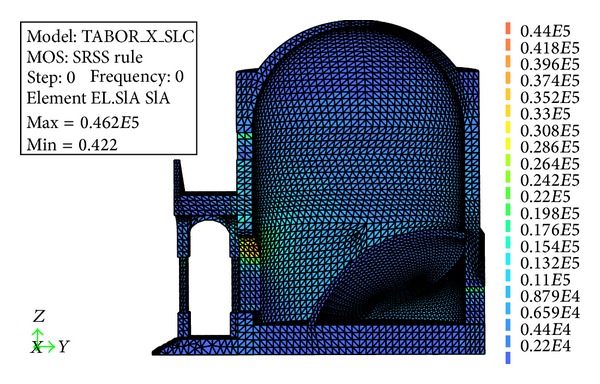
Chapel XVII principal SRSS stress contour, measured in Pa, during simulated earthquake in the *X* direction.

**Figure 15:**
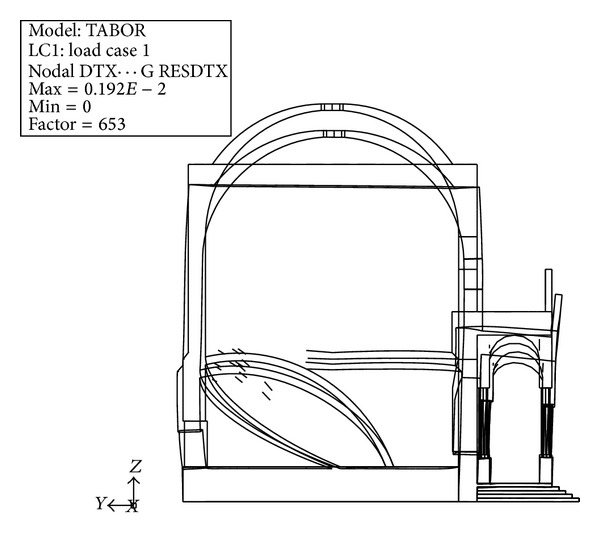
Chapel XVII deformed shape.

Figure 14 shows the contour of the principal tensile stress during simulated earthquake, reported on the deformed shape of the structure. The total combination of the seismic effects is performed using the Square Root of the Sum of the Squares (SRSS) method. The tensile stresses calculated on the internal wall of the chapel, subjected to dead loads and an earthquake in the *X* direction, justify the presence of the two symmetric dominant cracks detected by a visual inspection.  Figure 15 shows the deformed shape of the structure compared to the initial shape under the effect of the dead loads only. The deformation clearly shows the opening mechanism due to the effect of the pronao settlement, as well as to the thrust of the internal vault that supports the Mount Tabor installation.

The FEM analysis provides stress levels under dead loads near to the vertical cracks that are not so high for a stone masonry with mortar joints (see Figures 14 and 15). Therefore, the stability assessment derived by AE monitoring, reported in Section 2, is confirmed and a stable behavior of fractures is identified. On the other hand, the dynamic FEM analysis, in correspondence to the main seismic events, locally identifies levels of stress that agree well with the nucleation of microcracks after the arrival of the main seismic shockwave, which is detected as the AE aftershocks as described in Section 4.

A more detailed mechanical characterization of the masonry is currently under development to perform the subsequent nonlinear analysis.

## 6. Conclusions

Besides the canonical use in nondestructive tests, the heuristic potential of AE monitoring of civil structures for earthquakes prediction appears very intriguing. Starting from the assumption that any structure should not be regarded as separated from its environment, a method of correlating AE activity on the Renaissance Complex of the Sacred Mountain of Varallo subjected to a long-term monitoring with regional seismicity is investigated. Two qualitatively very similar phenomena such as Acoustic Emission and earthquakes become two aspects of a unique phenomenon, which looks self-similar.

Furthermore, in this work, by applying the modified Grassberger-Procaccia correlation algorithm, with the aim of explaining the correlation between regional seismicity and Acoustic Emission emerging from the Chapel XVII of the Sacred Mountain of Varallo, it is observed that the structure behaves as sensitive receptors for earthquakes occurring within a radius of about 100 km, distinguishing environmental contributions to AE activity on the Chapel XVII due to crustal trembling (external source) from contributions due to structural damage (inner source). An accurate finite element model, performed with DIANA finite element code for the dynamic analysis of Chapel XVII structure, is utilized to confirm visual inspections and monitoring the results of the earthquakes' effects.

## Figures and Tables

**Table 1:** AE as precursor from May 9, 2011 to June 16, 2011. Cumulative probability *C* ^+^(*r*, *τ*) for radius *r* ranging between 60 and 100 km, and time *τ* varying from 1 week to 5 weeks.

	60 km	80 km	100 km
1 week	0.0339	0.1121	0.2018
2 weeks	0.0772	0.2130	0.3661
3 weeks	0.1228	0.3018	0.4875
4 weeks	0.1487	0.3661	0.5549
5 weeks	0.1630	0.4321	0.6210

**Table 2:** AE as aftershock from May 9, 2011 to June 16, 2011. Cumulative probability *C* ^−^(*r*, *τ*) for radius *r* ranging between 60 and 100 km, and time *τ* varying from 1 week to 5 weeks.

	60 km	80 km	100 km
1 week	0.0254	0.0732	0.1437
2 weeks	0.0357	0.1196	0.2629
3 weeks	0.0371	0.1509	0.3362
4 weeks	0.0371	0.1652	0.3732
5 weeks	0.0371	0.1665	0.3768

**Table 3:** AE as precursor from July 5, 2011 to September 5, 2011. Cumulative probability *C* ^+^(*r*, *τ*) for radius *r* ranging between 60 and 100 km, and time *τ* varying from 1 week to 9 weeks.

	60 km	80 km	100 km
1 week	0.0075	0.0278	0.0846
2 weeks	0.0184	0.0552	0.1896
3 weeks	0.0239	0.0833	0.3222
4 weeks	0.0346	0.1040	0.3841
5 weeks	0.0498	0.1210	0.4435
6 weeks	0.0557	0.1268	0.5130
7 weeks	0.0557	0.1268	0.5497
8 weeks	0.0557	0.1268	0.5607
9 weeks	0.0557	0.1268	0.5657

**Table 4:** AE as aftershocks from July 5, 2011 to September 5, 2011. Cumulative probability *C* ^−^(*r*, *τ*) for radius *r* ranging between 60 and 100 km, and time *τ* varying from 1 week to 9 weeks.

	60 km	80 km	100 km
1 week	0.0045	0.0298	0.1192
2 weeks	0.0132	0.0465	0.1916
3 weeks	0.0234	0.0717	0.2592
4 weeks	0.0301	0.0970	0.3251
5 weeks	0.0313	0.1114	0.3737
6 weeks	0.0313	0.1246	0.4164
7 weeks	0.0313	0.1299	0.4283
8 weeks	0.0313	0.1336	0.4333
9 weeks	0.0313	0.1341	0.4338

**Table 5:** Filtered AE as precursor from July 5, 2011 to September 5, 2011. Cumulative probability *C* ^+^(*r*, *τ*) for radius *r* ranging between 60 and 100 km, and time *τ* varying from 1 week to 9 weeks.

	60 km	80 km	100 km
1 week	0.0080	0.0290	0.0829
2 weeks	0.0197	0.0592	0.1969
3 weeks	0.0252	0.0866	0.3288
4 weeks	0.0364	0.1081	0.3920
5 weeks	0.0517	0.1248	0.4538
6 weeks	0.0577	0.1308	0.5267
7 weeks	0.0577	0.1308	0.5625
8 weeks	0.0577	0.1308	0.5731
9 weeks	0.0577	0.1308	0.5776

**Table 6:** Filtered AE as aftershocks from July 5, 2011 to September 5, 2011. Cumulative probability *C* ^−^(*r*, *τ*) for radius *r* ranging between 60 and 100 km, and time *τ* varying from 1 week to 9 weeks.

	60 km	80 km	100 km
1 week	0.0042	0.0294	0.1149
2 weeks	0.0117	0.0464	0.1889
3 weeks	0.0203	0.0681	0.2521
4 weeks	0.0278	0.0935	0.3142
5 weeks	0.0292	0.1074	0.3604
6 weeks	0.0292	0.1202	0.4041
7 weeks	0.0292	0.1253	0.4151
8 weeks	0.0292	0.1292	0.4213
9 weeks	0.0292	0.1297	0.4218

**Table 7:** Natural frequencies of the first 20 calculated modes of vibration of Chapel XVII and percentages of mass involved in each mode of vibration for directions *X* and *Y* in the horizontal plane.

Mode	Frequency (Hz)	Period (s)	*X* direction		*Y* direction	
			%	Cumulated%	%	Cumulated%
1	4.737	0.211	5.31*E* + 01	5.31*E* + 01	5.89*E* − 05	5.89*E* − 05
2	5.162	0.194	5.82*E* − 05	5.31*E* + 01	4.97*E* + 01	4.97*E* + 01
3	8.570	0.117	5.94*E* − 02	5.32*E* + 01	2.61*E* − 07	4.97*E* + 01
4	9.891	0.101	1.20*E* − 01	5.33*E* + 01	1.43*E* − 05	4.97*E* + 01
5	10.087	0.099	1.50*E* − 05	5.33*E* + 01	1.95*E* − 01	4.99*E* + 01
6	13.192	0.076	3.37*E* + 00	5.67*E* + 01	4.59*E* − 06	4.99*E* + 01
7	13.373	0.075	1.68*E* − 04	5.67*E* + 01	2.85*E* − 01	5.02*E* + 01
8	13.881	0.072	6.60*E* + 00	6.33*E* + 01	5.39*E* − 06	5.02*E* + 01
9	14.479	0.069	6.48*E* − 05	6.33*E* + 01	9.44*E* + 00	5.96*E* + 01
10	14.616	0.068	1.13*E* − 04	6.33*E* + 01	3.83*E* + 00	6.35*E* + 01
11	15.633	0.064	2.65*E* − 05	6.33*E* + 01	3.31*E* − 01	6.38*E* + 01
12	17.402	0.057	2.36*E* − 02	6.33*E* + 01	5.14*E* − 06	6.38*E* + 01
13	17.612	0.057	3.66*E* − 06	6.33*E* + 01	5.25*E* − 02	6.38*E* + 01
14	18.031	0.055	5.02*E* − 01	6.38*E* + 01	1.91*E* − 05	6.38*E* + 01
15	18.677	0.054	2.45*E* − 04	6.38*E* + 01	1.28*E* − 01	6.40*E* + 01
16	18.750	0.053	9.08*E* − 02	6.39*E* + 01	1.67*E* − 04	6.40*E* + 01
17	19.165	0.052	6.71*E* − 02	6.40*E* + 01	1.91*E* − 05	6.40*E* + 01
18	19.874	0.050	3.13*E* − 09	6.40*E* + 01	2.02*E* − 01	6.42*E* + 01
19	20.545	0.049	4.58*E* − 01	6.44*E* + 01	7.58*E* − 06	6.42*E* + 01
20	21.578	0.046	5.79*E* − 06	6.44*E* + 01	5.48*E* − 01	6.47*E* + 01
